# Automated analysis of acute myeloid leukemia minimal residual disease using a support vector machine

**DOI:** 10.18632/oncotarget.12430

**Published:** 2016-10-04

**Authors:** Wanmao Ni, Beili Hu, Cuiping Zheng, Yin Tong, Lei Wang, Qing-qing Li, Xiangmin Tong, Yong Han

**Affiliations:** ^1^ Key Laboratory of Tumor Molecular Diagnosis and Individualized Medicine of Zhejiang Province, Zhejiang Provincial People's Hospital, Hangzhou, Zhejiang, P. R. China; ^2^ Medical of College, Zhejiang University, Hangzhou, Zhejiang, P. R. China; ^3^ The Center Hospital of Wenzhou, Wenzhou, Zhejiang, P. R. China; ^4^ Department of Hematology, Shanghai General Hospital, Shanghai, P. R. China

**Keywords:** flow cytometry, immunophenotyping, support vector machine, acute myeloid leukemia, minimal residual disease

## Abstract

We investigated the ability of support vector machines (SVM) to analyze minimal residual disease (MRD) in flow cytometry data from patients with acute myeloid leukemia (AML) automatically, objectively and standardly. The initial disease data and MRD review data in the form of 159 flow cytometry standard 3.0 files from 36 CD7-positive AML patients in whom MRD was detected more than once were exported. SVM was used for training with setting the initial disease data to 1 as the flag and setting 15 healthy persons to set 0 as the flag. Based on the two training groups, parameters were optimized, and a predictive model was built to analyze MRD data from each patient. The automated analysis results from the SVM model were compared to those obtained through conventional analysis to determine reliability. Automated analysis results based on the model did not differ from and were correlated with results obtained through conventional analysis (correlation coefficient c = 0.986, *P* > 0.05). Thus the SVM model could potentially be used to analyze flow cytometry-based AML MRD data automatically, objectively, and in a standardized manner.

## INTRODUCTION

Acute myeloid leukemia (AML) is the most common form of leukemia in adults. Moreover, it has the lowest survival rate of any type of leukemia [1]. During AML treatment, detection of minimal residual disease (MRD) using real-time quantitative polymerase chain reaction (RQ-PCR) and flow cytometry (FCM) provide a powerful basis for adjusting the diagnosis and treatment [2–8]. The presence of MRD is also strongly associated with risk of relapse and prognosis [9, 10]. RQ-PCR-based MRD detection is highly sensitive, but it depends on AML patients expressing specific fusion genes (e.g. AML1/ETO) or mutant genes (e.g. c-kit mutant), or overexpressing certain genes (e.g. Wilm's oncogene) [11–14]. Consequently, detection is limited. FCM can detect leukemia associated immunophenotyping (LAIP) in 70%–75% of AML patients' at an initial stage. The sensitivity of FCM is about 10^−4^ for MRD in AML whose immunophenotyping is obviously different from the normal cells, whereas the sensitivity is only 10^−3^ when immunophenotyping is partially overlapped with normal cells [15, 16].

Conventional FCM-based MRD detection has several disadvantages. (1) It requires LAIP, so more antibodies are consumed, though the detection schemes can be standardized [16]. (2) Because it requires the analysts to have enough experience with the antigen differentiation regularity to rule out reactive hyperplasia of normal cells, different analysts may draw different conclusions from the same data, making the results subjective. (3) It mainly uses a scatter diagram in two-dimensional space, so multidimensional FCM data are not completely utilized, and multidimensional space recognition cannot be carried out.

Support vector machines (SVMs) are supervised learning algorithms used with neural networks, and have been widely used for analysis of DNA microarray data [17, 18] and facial recognition [19, 20], among other applications. An SVM can simultaneously learn all of the features derived from definitively classified training data in multidimensional space, build a recognition model for these data, and then recognize and classify unknown data using this model. With multidimensional recognition, the SVM model has greater accuracy and precision than one- or two-dimensional recognition. In recent years, SVMs have been gradually applied to FCM data analysis [21–27] – e.g. it can distinguish malignant lymphocytes from benign lymphocytes. Toedling et al. employed an SVM to detect residual disease in acute B lymphocytic leukemia with 99.78% sensitivity and 98.87% specificity [28]. The small and rapid LIBSVM library developed by Chang et al. is a mature and perfect class library for all SVM algorithms [29].

In the present study, the LIBSVM was introduced into the data analysis to overcome the disadvantages of FCM-based MRD detection. By combining SVM multidimensional training and recognition characteristics with the multidimensional advantages of FCM, MRD can be quantified more precisely, objectively and economically.

## RESULTS

### Patient characteristics

In this study, 159 AML data sets were selected from 36 patients, including 22 men and 14 women with an average age 42 years (range, 17 to 72 years). The distribution of AML subtypes included 10 M0, 3 M1, 15 M2, 6 M5, and 2 M6. The M3, M4, M7 and other subtypes seldom expressed CD7, so few cases were selected.

### The effect of training cell number

**Figure 1:**
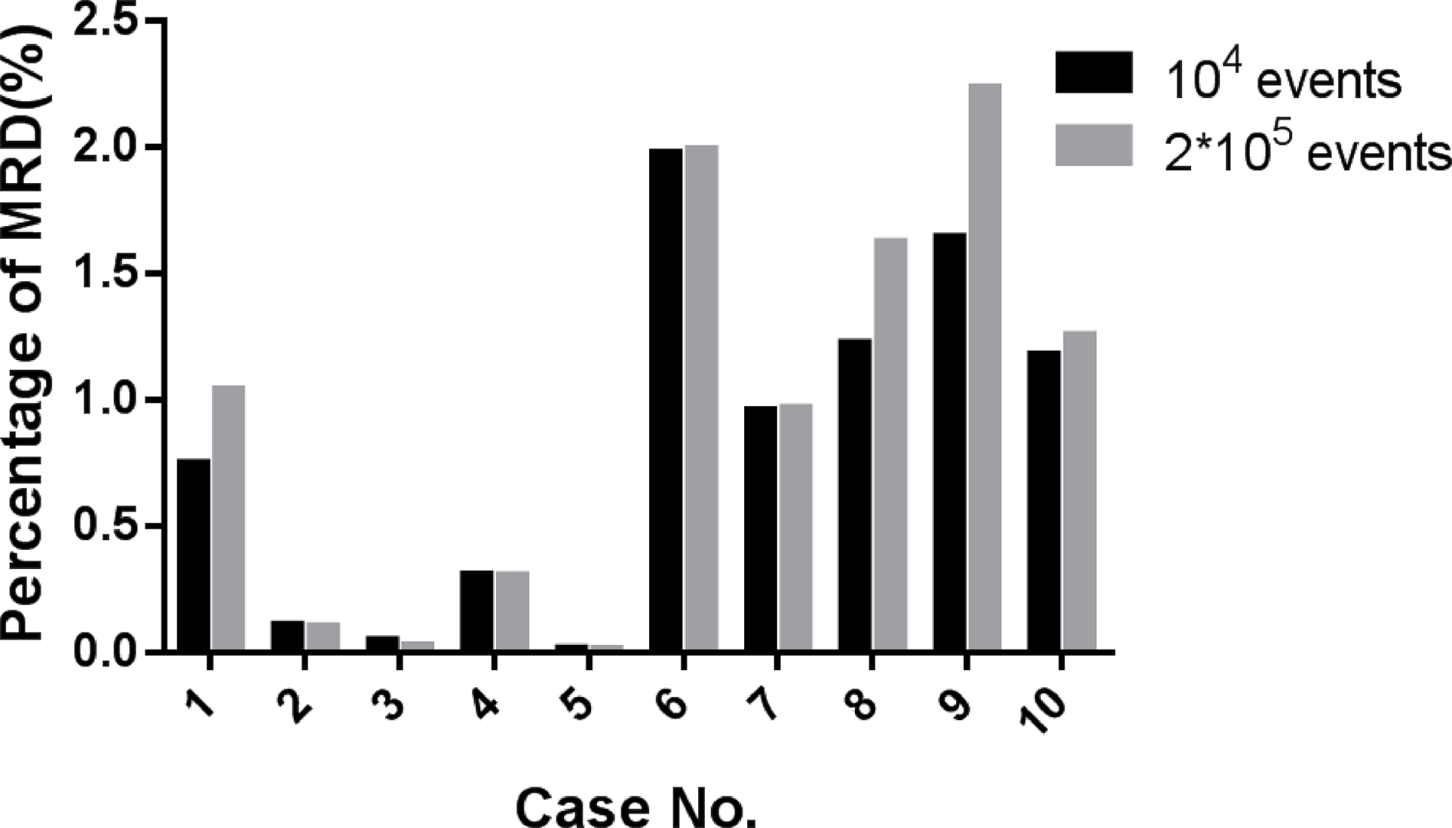
Comparison between different event numbers for training and calculating AML MRD (10^4^ events vs. 2 × 10^5^ events, *P* > 0.05)

Combing the data from the patients' initial immunophenotyping with the data from the 15 healthy individuals generated a training data file with more than 2 × 10^5^ events. However, because with LIBSVM the length of the training time was proportional to size of the training data file, using the complete training data set it took 24–36 h to finish the parameter optimization and model building with low efficiency. Therefore, to optimize parameters and model building more quickly, 10^4^ events were selected from the training data through stratified random sampling, which only took 5–10 min. In the patient group, the residual leukemic cell fractions in 10 AML MRD data sets were analyzed using the SVM. Notably, sets containing the 10^4^ sampled events did not significantly differ from the complete data sets (Paired *t*-test *P* = 0.0792) (Table 1 and Figure 1). Therefore, to improve the training efficiency, 10^4^ events selected through stratified random sampling were applied for training and model building.

**Table 1:** Comparison between different event numbers for training and calculating AML MRD

Case No.	10^4^ events	2 × 10^5^ events
**case 1**	0.747	1.037
**case 2**	0.108	0.101
**case 3**	0.047	0.024
**case 4**	0.305	0.304
**case 5**	0.013	0.012
**case 6**	1.974	1.986
**case 7**	0.956	0.965
**case 8**	1.222	1.621
**case 9**	1.640	2.230
**case 10**	1.174	1.253

### Optimization of training parameters C and γ

The grid.py script in LIBSVM was used to optimize parameters C and γ for each patient, based on the 10^4^ sampled event groups. Through optimization training for the 36 patients, 36 groups of C = 0.50–32768 (median, 8.00), γ = 0.13–8.00 (median, 8.00), and a corresponding optimized cross-validation accuracy of 98.25%–99.95% (median, 99.46%) were obtained, which meant that the accuracy of the individual-specific predictive models for the 36 patients could reach 98.25%–d99.95% when they were used in the MRD SVM analysis.

### Correlation between SVM group and manual group

**Figure 2:**
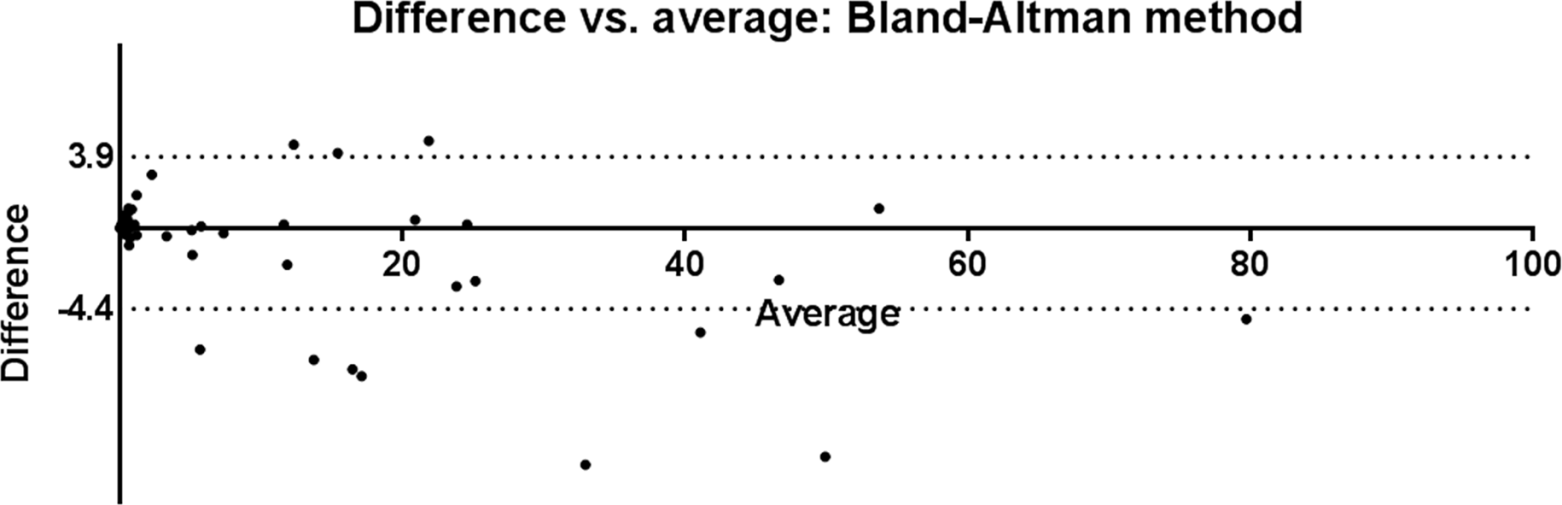
Bland-Altman comparison of SVM and manual analysis results Of the 159 pairs of data, only 11 were outside the 95% limits of agreement, which was from −4.4 to 3.9.

**Figure 3:**
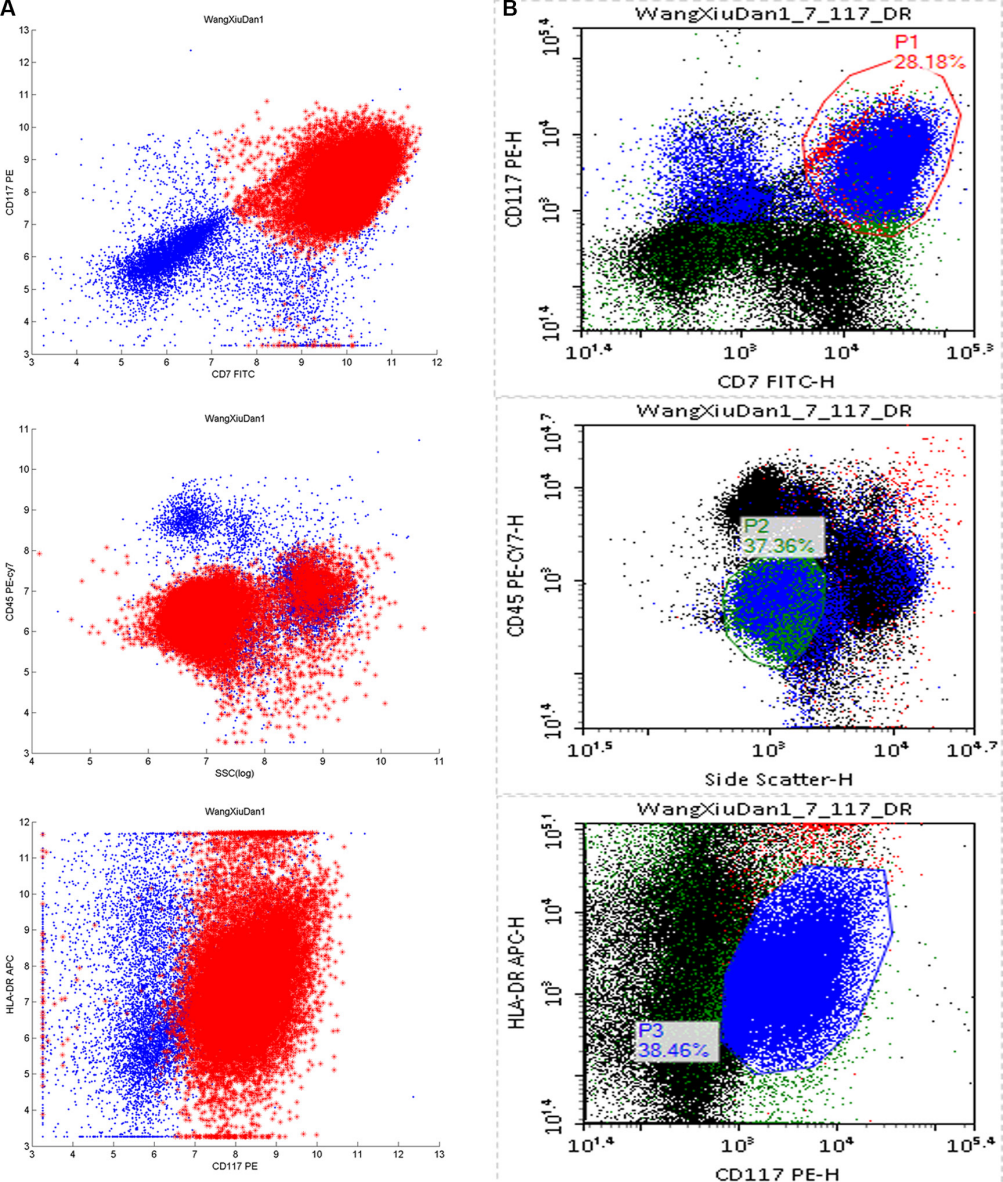
Comparison of the automatic SVM and manual analyses of typical AML patient results For clarity, each scatter diagram shows 10^4^ events. (**A**) The leukemic cell fraction was 24.672%, according to the SVM predictive model building of this MRD. The leukemia cell events are in red and the normal cells are in blue. (**B**) According to the manual analysis, the leukemia cell fraction was 24.466%, based on initial immunophenotyping of the patients. The gate was set by each step, and the MRD ratio was calculated as “P1 and P2 and P3”. The scatter diagrams were CD7/CD117, SSC/CD45, and CD117/HLA-DR, from top to bottom.

The 36 predictive models were used in the automated SVM analysis of MRD data, and the MRD data was also analyzed conventionally, yielding 159 groups of paired data. The MRD cell fraction was determined be 0.006%–82.180% using conventional analysis and 0.006%–77.200% using the automated analysis. The correlation coefficient was 0.986, and a two-tailed paired *t*-tests showed the correlation to be significant (*P* < 0.05) and without a significant statistical difference between the results (*P* = 0.134). Using the Bland-Altman comparison method, only 11 of the 159 data pairs were out of 95% limits of agreement (Figure 2). From the scatter diagrams, it was apparent that the distribution of leukemia cells determined using SVM analysis was similar to that obtained using manual analysis (Figure 3).

## DISCUSSION

An SVM is a supervised learning algorithm that can learn the characteristics of known objects in multiple dimensions then build predictive models with which to classify data of unknown classification [26]. LIBSVM is an excellent, easy and mature library. For FCM data, SVM has the advantage of being capable of multidimensional analysis, especially for 4 or more colors, and avoids the artificial misjudgment and experience requirements of an analyst. In this study, when LIBSVM was applied for automated MRD analysis, the results did not significantly differ from those obtained conventionally, and the results of the analysis could be displayed in different colors on scatter diagrams [29].

While the SVM is learning the known classification data and model building, parameter optimization can affect the ability of the model to accurately analyze unclassified data. C and γ are both important parameters for optimization [30]. C is the penalty coefficient, which controls the model's ability to generalize. If C is too large or too small, the ability of values to float will be poor [31]. Parameter γ controls the degrees of freedom in the nonlinear model – i.e. the number of support vectors. Only when C and γ are optimum does the model have the highest prediction accuracy (CV rate). Each patient expressed different levels of CD7, CD117, CD45, and HLA-DR, so the distributions differed in the multidimensional space. Consequently, when leukemia cells were mixed with normal cells to form the training data file, the dividing plane between the leukemic and normal cells differed in the multidimensional space. Thus, finding the corresponding optimal dividing plane for the patient, which would identify the optimal parameters of the individual-specific C and γ, was the key to establishing the SVM model [32]. The grid.py script in the LIBSVM class library was therefore used to optimize the parameters. However, all the data required cross-validation 5 times, and C and γ was tested step by step, so the optimization time and data volume were closely related. We found that if parameter optimization and model building were done using the complete training data (derived from patients with initial leukemia cells and 15 cases of normal data, including more than 20 million events), it would take 24–36 h to complete, which is not suitable for practical application. We therefore used a stratified random method to extract 10^4^ data as training data. Comparison with the automatic analysis results obtained with MRD models using two different size data sets revealed no significant difference between the models (*P* > 0.05).

When the optimized parameters were used to establish a predictive model, the results of the SVM automated MRD analysis did not differ from those obtained using the manual method (*P* > 0.05), with a correlation coefficient of 0.986. By comparing the results obtained using the SVM with those obtained manually from scatter diagrams, we found that the SVM properly studied the distribution characteristics of the patient's initial leukemia cells in multiple dimensions and accurately identified the residual leukemia cells.

In conclusion, automated SVM and manual analyses showed good consistency, which could reduce the experience requirements for MRD analysis. However, the sample size of AML patients involved was not large enough, and additional data from CD7-positive AML patients is needed. Moreover, AML patients exhibiting other expression patterns (e.g. CD19 expression without CD7 expression or abnormal CD33 and CD13 expression patterns without CD7 expression) should also be analyzed using an SVM in order to determine the scope of its utility. In short, the introduction of LIBSVM and other SVM algorithm libraries can be completely applied to the multidimensional features of FCM data, which would make interpretation of FCM data more objective and would provide a basis for achieving automated FCM data analysis.

## MATERIALS AND METHODS

### Patients

Immunophenotypes vary among AML patients, and there is no consensus on the LAIP for MRD detection. We studied one specific LAIP containing CD7, CD117, CD45 and HLA-DR. Information with the features listed below for patients treated from 2010 to 2012 were selected from the FCM database in our division. (1) Initial immunophenotyping schemes contained CD7, CD117, CD45 and HLA-DR, and these four antigens were detected in the same tube. (2) Leukemia residual disease lesion detection was carried out no less than twice. (3) The specimens were bone marrow. (4) The cell counts in the data file were at least 10^5^. Using these criteria, 159 data files from 36 patients were selected. An individual-specific predictive model was built for each patient.

### Reagents and instruments

CD7 (Becton Dickinson Biosciences, USA) was labeled with fluorescein isothiocyanate (FITC) using clone No. 4H9. CD117 (Beckman Coulter, Inc. USA) was labeled with phycoerythrin (PE) using clone No. 95C3. HLA-DR (Becton Dickinson Biosciences, USA) was labeled with allophycocyamin (APC) using clone No. G46-6. CD45 (Becton Dickinson Biosciences, USA) was labeled with phycoerythrin cyanin 7 (PE-cy7) using clone No. J33.

A BD FACS Calibur equipped with two lasers and four colors was applied for the MRD study. LIBSVM version 3.16 was used for model building, parameter optimization and data prediction for automated analysis. ACEA NovoExpress^TM^, which was developed by ACEA Bioscience Inc. to have a humanization design interface and functions, was used for manual analysis.

### Automated MRD analysis using LIBSVM

The individual model for each patient was built through the processes of training data file derivation, parameter optimization, and individual model building.

ACEA NovoExpress^TM^ was used to open the flow cytometry standard (FCS) data files for patients' initial immunophenotyping. The leukemia cells at diagnosis were selected and set as P1, after which P1 were exported in the comma separated value (CSV) file format. Thereafter, CSV files were imported into MATLAB R2011a and saved as matrices in which the columns were fluorescence, rows were cells, and the matrix values were fluorescence intensities. The matrix data were written into training data files named [patientname.train.txt] in the form required for LIBSVM, and the data flag was set as 1. FCS data from 15 healthy individuals were read in the same way, and the data were added to the aforementioned [patientname.train.txt] file. That data flag was set as 0. To normalize the data and speed up model building, ‘svm-scale.exe' was used to adjust the data in the [patientname.train.txt] file following the LIBSVM requirements so as to construct the [patientname.train.scale] file. At the same time, the [patientname.train.range] file was formed to enable adjustment of the data in the prediction stage.

After the data were ready, the LIBSVM default kernel (radial basis function) was employed to build individual-specific models. In addition, using the penalty coefficient C and core parameter γ, model prediction was optimized to form the optimized model. For this purpose, grid.py in LIBSVM was called to carry out the cross-validation of the training data file formed above. The default parameter values were set through 5-time cross-validation with the command ‘grid.py –svmtrain “svmtrain.exe” -log2c -gnuplot “gnuplot.exe” “[patientname.train.scale]”’. The optimized C and γ were obtained to build the patients' individual-specific training model with the command ‘svm-train.exe–c–g “[patientname.train.scale]” “[patientname.train.model]”’.

For the automated MRD analysis, the original data for a patient's MRD were processed in the standard LIBSVM form and a data flag was set to 1, as mentioned above. To recognize MRD data with different detection times, the file was named [patientname+detectiontime.test], and the aforementioned [patientname.train.range] file was used to scale [patientname+detectiontime.test] into [patientname+detectiontime.test.scale]. The svm-predict.exe program in the LIBSVM software package was then combined with the individual patient's specific training model to automatically analyze the [patientname+detectiontime.test.scale] file and predict the accuracy of flag 1; that is, the accuracy of the patient's residual leukemic cell fraction. Finally, the predicted leukemic cells (in red) and normal cells (in blue) were plotted using the “scatter” function in MATLAB.

### Conventional manual analysis of MRD

The three scatter diagrams CD7/CD117, SSC/CD45 and FSC/HLA-DR were plotted for patients' initial immunophenotyping. The P1, P2 and P3 gates were set for leukemia cells in the scatter diagrams. Finally, the leukemic cell fraction was obtained through the logical combination “P1 AND P2 AND P3”.

### Statistics

The data groups in the automated SVM analysis were compared to the corresponding data in the conventional manual analysis, one by one, using two-tailed paired *t* tests. Values of *P* < 0.05 were considered significant.
